# Comprehensive end-of-life care practices for older patients with heart failure provided by specialized nurses: a qualitative study

**DOI:** 10.1186/s12877-023-04050-6

**Published:** 2023-06-05

**Authors:** Mana Doi, Yukie Maruyama, Akiko Kaneda, Maya Minamizaki, Masami Fukada, Yuka Kanoya

**Affiliations:** 1 Department of Gerontological Nursing, Nursing Course, School of Medicine, Yokohama City University, 3-9 Fukuura Kanazawa-Ku, Yokohama, Kanagawa, 236-0004 Japan; 2 Tokyo Healthcare University, Chiba Faculty of Nursing, 1-1042-2 Kaijincho-Nishi, Funabashi, Chiba 273-8710 Japan

**Keywords:** Nurse specialists, End-of-life care, Heart failure, Older adults, Palliative care, Qualitative research

## Abstract

**Background:**

The context of end-of-life care for older heart failure patients with a complex clinical course provided by certified nurse specialists in gerontology (GCNSs) and Certified nurses in chronic heart failure (CNCHFs) is unclear; therefore, this study aims to describe comprehensive nursing practice for older patients with heart failure at their end of life.

**Methods:**

This study adopts a qualitative descriptive design using content analysis. Five GCNSs, and five CNCHFs were interviewed using a web app from January to March 2022.

**Results:**

Thirteen categories of nursing practices for older patients with heart failure were generated: (1) Provide thorough acute care by a multidisciplinary team to alleviate dyspnea, (2) Assess psychiatric symptoms and use a suitable environment to perform treatment, (3) Explain the progression of heart failure with the doctor, (4) Build a trusting relationship with the patient and family and implement advance care planning (ACP) early during the patient's recovery, (5) Involve multiple professions to help patients to achieve their desired life, (6) Perform ACP always in collaboration with multiple professionals, (7) Provide lifestyle guidance according to patients' feelings so that they can continue living at home after discharge from the hospital, (8) Provide palliative and acute care in parallel with multiple professions, (9) Achieve end-of-life care at home through multidisciplinary cooperation, (10) Provide basic nursing care to the patient and family until the moment of death, (11) Provide concurrent acute and palliative care as well as psychological support to alleviate physical and mental symptoms, (12) Share the patient's prognosis and future wishes with multiple professionals, and (13) Engage in ACP from early stages, through several conversations with patients and their families.

**Conclusions:**

Specialized nurses provide acute care, palliative care, and psychological support to alleviate physical and mental symptoms throughout the different stages of chronic heart failure. In addition to nursing care by specialized nurses at each stage shown in this study, it is important to initiate ACP early in the end-of-life stage and to provide care for patients with multiple professionals.

## Background

Globally, there are approximately 26 million heart failure patients [1]. In Japan’s super-aging society, the number of older patients with heart failure aged 65 years and above is expected to increase and reach 350,000 by 2030 [2]. Heart failure among older adults is caused by various conditions such as ischemic heart disease or valvular disease. Furthermore, this condition gradually worsens with readmissions following acute exacerbations. In case of this acute exacerbation, including newly ischemic heart disease and lethal arrhythmia, sudden death could occur [3]. In fact, it is reported that heart failure patients are hospitalized once every year on average after their initial diagnosis; additionally, 1-year mortality rates after diagnosis reached 20% [4]. This tendency is remarkable in older patients. Consequently, the early introduction of palliative care and decision-making support for older adults has been emphasized. Moreover, medical professionals in cardiovascular medicine are required to provide both acute and end-of-life care based on a multifaceted approach including palliative care.

The end-of-life care policy has been underscored in European Society of Cardiology Guidelines of Heart Failure in older adults [4], which discusses the ideal system for providing palliative care for patients with cardiovascular diseases. In such an ideal system, specialized nurses, that is, certified nurse specialists (CNSs) and certified nurses in Japan, are expected to play a crucial role [5]. In America and Europe, heart failure nurses (HF nurses) manage the symptoms of heart failure patients, sometimes as part of a team [3, 4, 6, 7]. It has been reported that doctors [6] prefer a more extensive approach; thus disease-management would be a primary role for them. However, in Japan, a super-aging society, certified nurses in chronic heart failure (CNCHFs) are expected to not only manage patients’ disease and support older patients with heart failure from hospitalization to post-discharge according to their lifestyles, but also support their life adjustment until their end-of-life period [8]. Furthermore, CNSs in gerontology (GCNSs) provide nursing care for older patients with complex health problems [9] by visiting various wards in their hospitals, not only the ward to which they belong. GCNSs care for older patients regarding their complex health problems and developmental issues during their end-of-life period when ethical considerations, such as decision-making support, are required, which is the focus of this research. Therefore, GCNSs and CNCHFs can provide multifaceted approaches involving family members in acute and palliative care and offer decision-making support required by older patients with heart failure. However, prior reports on the practice of CNSs and certified nurses caring for older patients with heart failure focused primarily on a single aspect, either palliative care or decision-making support approaches [5, 10]. Yet, nurses provide care from several aspects simultaneously.

Therefore, older patients with heart failure require end-of-life care based on a multifaceted approach. The American Heart Association indicated that frequent appraisals in a complex trajectory can guide patient-related communication and decision-making [3]. Thus, the approach provided by CNSs and certified nurses could be associated with patients’ trajectory. In addition to acute care, these specialists could also provide psychological support. However, the context of end-of-life care for older patients with chronic heart failure provided by GCNSs and CNCHFs has not been clarified. Therefore, this study aimed to describe the practice of comprehensive nursing for older patients with end-of-life heart failure performed by GCNSs and CNCHFs based on the clinical course of patients.

## Methods

### Aims and objective

This study aimed to describe the practice of comprehensive nursing (i.e., acute care, palliative care, decision-making support, and other multifaceted approaches) for older patients with end-of-life heart failure performed by GCNSs and CNCHFs in Japan based on the clinical course of patients.

### Design

Our study adopted a qualitative descriptive design using content analysis.

### Sampling and recruitment

The participants were GCNSs and CNCHFs certified by the Japanese Nurses Association who had experience in end-of-life care of older patients with heart failure. One of the researchers, AK, is a GCNS. She contacted available GCNSs in Japan regarding their possible participation in this study. We then informed them about the study details. We continued recruitment using snowball sampling. We concluded recruitment once the data reached saturation, i.e., at a point of no additional data provision regarding nursing practice [11].

### Exclusion criteria

GCNSs and CNCHFs with no experience in providing end-of-life care to this patient group were excluded. Furthermore, certified nurses in palliative care were excluded because they specifically focus on palliation of symptoms, such as pain and difficulty in breathing, and their main activities focus on cancer patients.

### Data collection

Semi-structured interviews via the Zoom application were conducted. A password and identification were created for the room on Zoom, which only permitted the participation of researchers and study participants. We conducted interviews lasting 40–60 min between January 26 and March 15, 2022, and recorded the interviews. To elicit narratives on nursing practices based on the patient’s clinical course, the interviews were conducted using the patient’s clinical course to guide the discussion [3]. Based on previous studies [5, 12], the researchers in this study established the following interview items: “In terms of the end-of-life period, please describe your usual involvement with older patients with heart failure;” “Please discuss any impressions you received while caring for older patients with heart failure during the end-of-life period;” “Focusing on the end-of-life period, please describe any nursing care that you provide consciously to older patients with heart failure;” “How do you discuss end-of-life and prognosis matters with patients and their families?;” “How do you conduct conversations on advance care planning (ACP), life-prolonging measures, and treatment options with older patients with heart failure?;” “How do you conduct discussions underscoring ACP, life-prolonging measures, and treatment options with the family?;” “Please explain how you manage patients’ symptoms such as dyspnea, pain, depression, anxiety, nausea, fatigue, and insomnia among others;” “Please describe how you provide psychological support such as listening to patients;” “Please describe how you implement care coordination through the use of home hospice and coordination of social resources;” “Please discuss the multidisciplinary collaboration (e.g., conferences) in relation to the process, its implementation, and any important points of collaboration or timing;” “How do you involve the patient’s family?;” and “Please share any thoughts or experiences about how you provide multiple types of care (e.g., acute and palliative care) at the same time or in combination.”

When participants did not mention which part of the clinical course they were referring to, this was confirmed by asking them whether they were currently at the stage of “the onset of chronic heart failure,” “decompensation,” “advanced chronic heart failure,” or a different timeframe. Moreover, we collected data on participants’ age, gender, education, place of work (e.g., hospital ward), years of clinical experience, years of experience in heart failure patient care, and years of certified experience as GCNS/CNCHF.

The current study defined “older adults” as individuals aged 65 years or older, while “end-of-life” refers to the last months or years of an individual’s life [13].

### Data analysis

Three methods of content analysis have been described by Hsieh and Shannon [14]. The current study used directed content analysis, which is based on an existing conceptual framework (theory or prior research). The data were analyzed based on the method of Elo and Kyngäs [15]: deciding what to analyze during preparation; open coding; creating categories; and abstraction, which continues as far as feasible. Then, we used the clinical course of the patients [3] as the basis for the analysis. Interviews were transcribed, and interview data indicating end-of-life care practices of GCNS/CNCHF were extracted. Subsequently, the data were classified and abstracted according to four categories: “the onset of chronic heart failure,” “decompensation,” “advanced chronic heart failure,” and “not bounded by time axis.”

The contents of the extracted data were reviewed and coded while ensuring the meaning was retained. Thereafter, the codes were abstracted into sub-categories and categories and named according to nursing practice, emphasizing the similarities and differences in meaning.

### Ethical considerations

Prior to participating in the study, the participants were fully informed about the study’s purpose, and consent was obtained in writing. This study was approved by Yokohama City University Ethics Committee (approval number: F211100036).

### Rigor

All interviews were conducted by the researcher (MD). Two researchers (MD, YM) analyzed the same data from coding to categorization and arrived at the same conclusion (Flick, 2022) [11]. In addition, to ensure the reliability of the data, the entire analysis process was reviewed by research group members with experience in the field of cardiology, nursing care of older patients, or qualitative research.

## Results

### Participants

Five GCNSs and five CNCHFs participated in this study. The characteristics of the participants are shown in Table 1. As a result of the analysis based on the course of Decision Making in Advanced Heart Failure [3], 218 codes, 45 sub-categories, and 13 categories were generated (Table 2). Thirteen categories of nursing practices were identified as follows: five in the period of the onset of chronic heart failure, two in decompensation, three in advanced chronic heart failure, and three in any period. Representative codes and quotes with participant’s number are described below (e.g., GCNS1 represents GCNS No.1. All participants are coded as GCNS No.1–5 and CNCHF No.1–5). These correspond to each category shown in Table 2.

**Table 1:** Characteristics of the participants

Participants	Age	Gender	Years of experience			Area of work	Educational level
			as nurse	in heart failure nursing	as GCNS or CNCHF		
GCNS1	50 s	Female	30–34	10–14	1–4	Division of nursing administration	Graduate school
GCNS2	30 s	Female	10–14	10–14	1–4	Cardiology	Graduate school
GCNS3	30 s	Male	10–14	10–14	1–4	General internal medicine	Graduate school
GCNS4	40 s	Male	15–19	1–4	1–4	Cardiology	Graduate school
GCNS5	50 s	Female	35–39	10–14	10–14	Dementia ward	Graduate school
CNCHF1	50 s	Female	30–34	10–14	5–9	Cardiology	Junior college
CNCHF2	30 s	Male	15–19	15–19	5–9	Cardiology	Nursing school
CNCHF3	30 s	Female	15–19	5–9	1–4	High care unit	Junior college
CNCHF4	50 s	Female	30–34	25–29	5–9	Cardiology	Junior college
CNCHF5	50 s	Female	25–29	15–19	5–9	Cardiology	Junior college

*GCNS* Certified Nurse Specialists in Gerontology, *CNCHF* Certified Nurses in Chronic Heart Failure

**Table 2:** Nursing practices for end-of-life care: categories and sub-categories

Time period	Category (category no.)	Sub-category (sub-category no.)	Representative code
Onset of Chronic Heart Failure	Provide thorough acute care by a multidisciplinary team to alleviate dyspnea (1)	Ensure that the doctor directly assesses the patient for intervention (1a)	GCNS3
		Provide multidisciplinary responses to emergency patients (1b)	
		Respond to sudden critical changes in patients, using acute treatment and sedation (1c)	
		Provide acute treatment for palliation of dyspnea (1d)	GCNS1
	Assess psychiatric symptoms and use a suitable environment to perform treatment (2)	Incorporate the patient’s lifestyle and favorite items into the treatment environment to encourage treatment (2a)	GCNS1
		Assess psychiatric symptoms including delirium (2b)	GCNS2
	Explain the progression of heart failure with the doctor (3)	Ask the doctor to explain the patient’s medical condition, including the risk of sudden death (3a)	CNCHF2
		Explain the process of heart failure carefully several times to ensure the patient’s understanding (3b)	CNCHF2
	Build a trusting relationship with the patient and family and implement ACP early during the patient’s recovery (4)	Implement ACP early, considering the patient’s background (4a)	GCNS3
		Build a trusting relationship with the patient and family, and use the outpatient clinic to discuss ACP after confirming the patient is stable (4b)	
		Communicate the desire to align with the patient’s feelings before introducing ACP (4c)	
		Confirm the patient’s wishes for future life and treatment and share information among professionals once the patient has stabilized (4d)	
		Provide an opportunity to consider ACP using tools for its implementation (4e)	
	Involve multiple professions to help patients to achieve their desired life (5)	Provide life-supporting interventions in parallel with acute care by multidisciplinary staff (5a)	GCNS2
		Adopt system innovations to ensure that multidisciplinary conferences are held as required (5b)	
		Discuss with staff when there is a gap between the future intentions of the patient and their family, and liaise between them to address such gaps (5c)	
		Coordinate discharge by multidisciplinary staff to realize the post-discharge life that the patient desires (5d)	GCNS3
Decompensation	Perform ACP always in collaboration with multiple professionals (6)	Recognize when to confirm the patient’s wishes regarding treatment and the future to establish a collaboration with the nurses (6a)	GCNS2
		Reconfirm ACP at readmission, ensuring the patient who has already confirmed ACP is not burdened (6b)	
		Work with multiple professionals to communicate prospects, confirm where the patient would like to spend the end of their life, and ensure that the patient’s and family’s wishes are met (6c)	
		Engage in dialogue to elicit the patient’s wishes (6d)	
	Provide lifestyle guidance according to patients’ feelings so that they can continue living at home after discharge from the hospital (7)	Inform the patients of the criteria for outpatient hospital admittance to determine if a patient prefers to be treated at recurrence (7a)	GCNS3
		Provide lifestyle and rehabilitation guidance gradually by mitigating the patient’s suffering and pain (7b)	
		Provide necessary information to patients who wish to remain at home and work with them to determine the cause of rehospitalization (7c)	CNCHF4
Advanced Chronic Heart Failure	Provide palliative and acute care in parallel with multiple professions (8)	Discuss the need for palliative care with doctors (8a)	CNCHF1
		Inform the family about palliative care using narcotics and initiate palliative care through collaboration with a palliative physician and team (8b)	
		Use a combination of palliative and acute care for symptom palliation (8c)	CNCHF2
		Explain to patients, family, and staff the need for concurrent palliative and acute care (8d)	
	Achieve end-of-life care at home through multidisciplinary cooperation (9)	Explain the current situation to the patient and family and confirm the patient’s wishes for end-of-life care at home (9a)	GCNS1
		Realize end-of-life care at home by alleviating symptoms through multidisciplinary cooperation (9b)	CNCHF1
	Provide basic nursing care to the patient and family until the moment of death (10)	Provide basic care and support to ensure that the patient can live as he/she wishes until the moment of death (10a)	GCNS2
		Provide nursing care for the family, for example, by conveying the importance of taking care of the family’s health (10b)	GCNS1
Any Period	Provide concurrent acute and palliative care as well as psychological support to alleviate physical and mental symptoms (11)	Provide acute treatment and palliative care for symptom relief (11a)	CNCHF2
		Respond to the patient’s pain and fears by listening, bathing the feet, walking, and improving the environment (11b)	
		Consult with psychiatry-related professionals and teams and provide mental and spiritual support (11c)	
	Share the patient’s prognosis and future wishes with multiple professionals (12)	Involve patients who have heart failure and need NPPV from the time of hospitalization, or who are recognized by a physician that the patient needed further consultation with GCNSs/CNCHFs (12a)	GCNS5
		Understand the patient’s future and intentions through conferences with various professional (12b)	
		Share the patient’s wishes among nurses and collaborate with the physician to realize them (12c)	
		Share with other nurses and practice the future outlook and policies of nursing care (12d)	
		Connect patients with multi-professionals to make home care possible by focusing on the patient’s life wishes after discharge from the hospital (12e)	
		Provide patients and families with lifestyle guidance to maintain life after discharge by a multidisciplinary team including the outpatient department (12f)	
	Engage in ACP from early stages through several conversations with patients and their families (13)	Perform ACP as early as possible before the condition transitions to advanced heart failure (13a)	GCNS4
		Capture the topic of conversation with the patient including what the patient says and the atmosphere before linking it to ACP (13b)	
		Approach the patient in a timeous manner until the patient adequately understands their medical condition (13c)	
		Confirm the thoughts and wishes of patients with their families and work collectively to realize them (13d)	CNCHF2

*ACP* Advance Care Planning, *CNCHF* Certified Nurses in Chronic Heart Failure, *GCNS* Certified Nurse Specialists in Gerontology, *NPPV* Non-invasive Positive Pressure Ventilation

### Nursing during the onset of chronic heart failure

#### Provide thorough acute care by a multidisciplinary team to alleviate dyspnea

This category comprises four sub-categories: (1a) Ensure that the doctor directly assesses the patient for intervention; (1b) Provide multidisciplinary responses to emergency patients; (1c) Respond to sudden critical changes in patients, using acute treatment and sedation; and (1d) Provide acute treatment for palliation of dyspnea.

Multidisciplinary staff, including specific nurses, certified intensive care nurses, and physicians from other departments, are involved in patient suffering and emergencies (code GCNS3). At the beginning of the acute phase, specialized nurses use NPPV (non-invasive positive pressure ventilation), intravenous hyperalimentation (IVH), and insert an A-line for thorough management (code GCNS1).

“After all, in the acute phase, in heart failure now, in the acute phase such as in chronic heart failure, the medical aspect will inevitably become large, so I will involve a nurse practitioner. Also, there are certified nurses for intensive care, so I consult with them regarding intensive care. Fortunately, our hospital doesn’t have to go through the doctor, so if you see something different, you see something strange, or you feel that you want someone to have a look and need help, then you can consult with intensive care or a specific nurse. I usually ask another nurse to take a look, right now. Also, I report to the doctor about the patient hoping that the doctors will take a look at the patient. There are some doctors who just call and ask me if they can follow up without having a look. I’d always tell them to come once, or whoever can come at least. If it doesn’t work, then I consult with other doctors, or if there is a registrar, then I would ask them to come and have a look, and we even ask the registrar to ask the doctors to come and see the patient in person.” (GCNS3).

“(Regarding palliative care at the beginning of the acute phase) It’s a very low priority, to be frank. I’m ashamed to say that I’m far from palliative care, because we have to use NPPV even if we use physical restraint, and we have to put in a continuous infusion of IVH, and we have to put in an A-line” (GCNS1).

#### Assess psychiatric symptoms and use a suitable environment to perform treatment

This category includes two sub-categories: (2a) Incorporate the patient’s lifestyle and favorite items into the treatment environment to encourage treatment and (2b) Assess psychiatric symptoms including delirium.

Specialized nurses put favorite objects in the patient’s room to motivate the patient who is still conscious to find hope after the hospital and as mental support to motivate the patient to engage in the treatment (code GCNS1). Also, it is important to be involved in adjusting their lives to prevent delirium and share with staff nurses because older adults are prone to delirium during the acute phase of chronic heart failure (code GCNS2).

“I want the patient to be full of strength to somehow recover, while suspecting that his/her anxiety or depression may be a low-activity type of delirium. For example, (there was) a woman who experienced slight depression, was still conscious, and loved Hawaiian music. Her family brought her muumuus. And I would put them on display and cheer her up by telling her that she’s going to dance again without oxygen.” (GCNS1).

#### Explain the progression of heart failure with the doctor

This category includes two sub-categories: (3a) Ask the doctor to explain the patient’s medical condition, including the risk of sudden death, and (3b) Explain the process of heart failure carefully several times to ensure the patient’s understanding.

Regardless of the severity of the illness, specialized nurses have the doctor explain the prognosis at the time of initial admission and talk about the possibility of sudden death (code CNCHF2). Furthermore, specialized nurses check the patient’s understanding of the doctor’s explanation of the condition and prognosis, and if there is any deficiency, they step in and discuss it according to the clinical course of the handbook issued by the academic society (code CNCHF2).

“I think it’s important to talk about ACP when being hospitalized for the first time. When a patient comes in with heart failure, even if it’s a mild or serious condition, even if it’s a 4^th^ or 2^nd^ degree of NYHA (New York Heart Association Functional Classification), the first step is an explanation by the doctor about ACP and prognosis. In fact, we talk about the trajectory of the disease, including the possibility of sudden death as well.” (CNCHF2).

“The doctor briefly talks to the patient about the prognosis. Then, I ask the doctor if I can give the patient more details about the prognosis, and he/she says he/she will leave it to me. I then tell the patient that the doctor just talked briefly about it, and we talk about it as we look at this disease track. The doctor also knows what the pamphlet (about the disease trajectory) says, of course, so as long as I follow it and talk about it, I won’t get in trouble.” (CNCHF 2).

#### Build a trusting relationship with the patient and family and implement ACP early during the patient’s recovery

This category includes five sub-categories: (4a) Implement ACP early, considering the patient’s background; (4b) Build a trusting relationship with the patient and family, and use the outpatient clinic to discuss ACP after confirming the patient is stable; (4c) Communicate the desire to align with the patient’s feelings before introducing ACP; (4d) Confirm the patient’s wishes for future life and treatment and share information among professionals once the patient has stabilized; and (4e) Provide an opportunity to consider ACP using tools for its implementation.

At a time when the patient has recovered a little, specialized nurses ask what they want to do in the future in terms of ACP and advanced directives, what kind of life they want, and whether they want acute treatment again at the time of recurrence (code GCNS3).

“When he/she gets a little better, this is exactly when I ask the patient about ACP and advanced directives (when the onset of chronic heart failure improves). I think whenever the patient is suffering, he/she cannot think of anything better, because they might have delirium, and it could be confusing from them being in the acute stage or something. Asking them about this might make them suffer more, that is why I usually try and start asking them about ACP when they get into recovery. I ask them what their life plan is and what kind of life they want at first in their room. Of course, the patient may or may not be on BiPAP, or may be off oxygen, but there is a possibility that the patient will suffer again in the future, so I also ask if he/she would like to have BiPAP again or not.” (GCNS3).

#### Involve multiple professions to help patients to achieve their desired life

This category consists of four sub-categories: (5a) Provide life-supporting interventions in parallel with acute care by multidisciplinary staff; (5b) Adopt system innovations to ensure that multidisciplinary conferences are held as required; (5c) Discuss with staff when there is a gap between the future intentions of the patient and their family, and liaise between them to address such gaps; and (5d) Coordinate discharge by multidisciplinary staff to realize the post-discharge life that the patient desires.

Specialized nurses provide rehabilitation and hygiene care in cooperation with various professions and prevent disuse syndrome in the acute stage of chronic heart failure so that patients can live after recovery (code GCNS2). Also, they involve care managers and social workers to coordinate with patients to achieve the post-discharge life they desire (code GCNS3).

“As I value nursing care for older patients, I want to make sure that their lives are not left behind. Even if an older patients is hospitalized in the acute stage, they are not just resting on the floor, but they need to be able to wake up in the morning and wash their face, eat properly, and engage in rehabilitation, etc. We need to be aware of the fact that they are able to live their lives. If the nursing involvement at this time is not appropriate or is neglected, the disuse syndrome will definitely progress, and although the heart is getting better, their physical condition will not be able to keep up. This time (when the acute deterioration of chronic heart failure is rapidly worsening) is when I think it is very important for doctors, rehabilitation staff, and pharmacists to work together, and I have been working on this recently.” (GCNS2).

“We also have a nurse and a social worker involved and ask what the family thinks about the patient who has such feelings but does not want to be transferred to a different hospital. For example, if a person wants to go home when he/she is about 80 or 90 years old, we usually involve the care manager as well. I also contact the care manager. I did it yesterday. I contact the care manager and say, ‘This is how the person wants to live, and he/she wants to go home. He/she wants to live like this, but his/her family is not cooperating with his/he care at all. So I would like the family to understand. The patient wants to live at home, so I ask you (the care manager) what I should do.’ Then, the care manager will make adjustments […], it is possible to bring in social resources so that the patient can go home, so we liaise with the care manager to coordinate with the patient’s family and the patient’s wishes, and then the patient is discharged.” (GCNS3).

### Nursing during decompensation

#### Perform ACP always in collaboration with multiple professionals

This category contains four sub-categories: (6a) Recognize when to confirm the patient’s wishes regarding treatment and the future to establish a collaboration with the nurses; (6b) Reconfirm ACP at readmission, ensuring the patient who has already confirmed ACP is not burdened; (6c) Work with multiple professionals to communicate prospects, confirm where the patient would like to spend the end of their life, and ensure that the patient’s and family’s wishes are met; and (6d) Engage in dialogue to elicit the patient’s wishes.

Specialized nurses inform the patient about the gradual decline in his/her condition, including the heart, together with the doctor (code GCNS2).

“The gradual decline is a process of repeated readmissions to the hospital, but it depends on the cognition and reserve capacity of older adults, and we need to educate patients. It may look as if things have returned to normal, but this does not mean that things are completely back to normal. It is shocking to know that the heart will continue to weaken little by little and will not be able to fully recover, but we have to cooperate with the doctors and inform patients of this.” (GCNS2).

#### Provide lifestyle guidance according to patients’ feelings so that they can continue living at home after discharge from the hospital

This category includes three sub-categories: (7a) Inform the patients of the criteria for outpatient hospital admittance to determine if a patient prefers to be treated at recurrence; (7b) Provide lifestyle and rehabilitation guidance gradually by mitigating the patient’s suffering and pain; and (7c) Provide necessary information to patients who wish to remain at home and work with them to determine the cause of rehospitalization.

Specialized nurses sympathize with and listen to the patient’s pain and anxiety about breathing difficulties at the time of acute deterioration and support the patient’s discharge home by providing lifestyle guidance including rehabilitation to prevent the patient from getting worse (code GCNS3). In addition, they involve patients in extending the time between patients’ next hospitalization, because once they are readmitted, they are often readmitted after a short period (code CNCHF4).

“I think that heart failure patients are very sensitive to breathing because they have experienced suffering at the onset of chronic heart failure. So I think that they have a strong feeling of what they should do if they suffer again, so I have to make sure that they follow the current treatment and rehabilitation. I don’t know how to say it, but I can say that the things that make him/her suffer are decreasing. It’s difficult. I’ll be there for their feelings, but… I can tell them, “I know how painful it was for you, how hard it was for you, but now you’re getting better.” I’m sure that’s all I can do. I know that there are people who are chronically depressed because of heart failure, and they think, “Who cares about me anyway?” I would talk to them about how they should do their best and give them psychological support so that they can go home as soon as possible” (GCNS3).

“There are so many older patients. Currently, there are many patients who are about 90 years old who come to the ICU (intensive care unit) for full treatment. It is generally known that readmission to the hospital once shortens the life expectancy of the patient. I would like to extend the time from the first readmission to the next readmission as much as possible.” (CNCHF4).

### Nursing during advanced chronic heart failure

#### Provide palliative and acute care in parallel with multiple professions

This category consists of four sub-categories: (8a) Discuss the need for palliative care with doctors; (8b) Inform the family about palliative care using narcotics and initiate palliative care through collaboration with a palliative physician and team; (8c) Use a combination of palliative and acute care for symptom palliation; and (8d) Explain to patients, family, and staff the need for concurrent palliative and acute care.

Specialized nurses provide care with the palliative care team using narcotics and other drugs (code CNCHF1); however, they make no particular distinction between palliative or aggressive treatment. Rather, the patient’s suffering and symptoms are addressed to alleviate them (code CNCHF2).

“The palliative care team mainly comes in for palliation of heart failure. The cardiac care team typically does not recognize the need for palliative care until this late stage, making it difficult for the palliative care team to intervene until the patient has entered the final stages of heart failure. At this stage the patient has already entered a stage of respiratory distress and cardiovascular drugs are not able to keep up, thus, morphine and other drugs are used.” (CNCHF1).

“In fact, it is difficult to know whether this is treatment or palliative care for older patients with heart failure, and I think this is still an issue. I talk with the doctors about whether it would be better to relieve the patient’s pain or to use dobutamine to relieve the symptoms of LOS (low cardiac output syndrome). But I think it is true that the doctors, myself, and the other staff on the ward do not have any particular ideas about what palliative care is and what active treatment is.” (CNCHF2).

#### Achieve end-of-life care at home through multidisciplinary cooperation

This category includes two sub-categories: (9a) Explain the current situation to the patient and family and confirm the patient’s wishes for end-of-life care at home and (9b) Realize end-of-life care at home by alleviating symptoms through multidisciplinary cooperation.

Patients and their families who are not sure if they want to be discharged to go home, including patients in the 20% ejection fraction (EF) range who are bedridden and barely able to eat, are able to receive end-of-life care at home through discharge coordination that takes into account their cardiac function and background (code GCNS1). Specialized nurses also work with the discharge support department and social worker to make it happen for those who wish to be discharged to go home (code CNCHF1).

“The patient will be 100 years old with his/her EF of 20%. And when he/she talks, he/she is in a lot of pain. So, he/she is already in bed and needs all the assistance he/she can get, and he/she can only eat at a level he/she enjoys, so it is the end-of-life stage for him/her. The family was very confused, but what we focused on was his/her 100^th^ birthday. I told them that it was not a dream for them to stay at home if they wanted to, and I also talked about the specifics of local medical care. The basis for this is really the patient’s cardiac function and his/her background, and the essence of the discharge coordination can support the family. For those who say they can’t go home, we will find an appropriate medical facility, but for those who are not sure, we work in that way. And then, generally, people say, ‘Well, I’m going to take him/her home.’” (GCNS1).

#### Provide basic nursing care to the patient and family until the moment of death

This category consists of two sub-categories: (10a) Provide basic care and support to ensure that the patient can live as he/she wishes until the moment of death and (10b) Provide nursing care for the family, for example, by conveying the importance of taking care of the family’s health.

Specialized nurses are involved to make sure that the patient can live their own life until the moment their life ends, whether the patient wants to go to the toilet by themselves until the end of their life or whether they would be more comfortable with a catheter (code GCNS2). In cases where end-of-life care is provided at the hospital, arrangements for the patient are made to spend time with his/her family in a comfortable environment, including the arrangement of a private room, and the family members are spoken to during rounds (code GCNS1).

“We think that it would be easier for the patient to use a catheter, but we also wonder what the patient would like to do. If it seems physically difficult for the patient to go to the toilet, or if he/she wants to go to the toilet until the end of his/her life, then we have to think about where we can find an alternative plan. We often say that patients are able to live their lives as they are until the moment of their life comes to an end, and I think that this will not be possible unless we look at this point properly.” (GCNS2).

### Nursing during any period

#### Provide concurrent acute and palliative care as well as psychological support to alleviate physical and mental symptoms

This category comprises three sub-categories: (11a) Provide acute treatment and palliative care for symptom relief; (11b) Respond to the patient’s pain and fears by listening, bathing the feet, walking, and improving the environment; and (11c) Consult with psychiatry-related professionals and teams and provide mental and spiritual support.

Specialized nurses consult with a psychiatrist or ask a home care nurse to provide mental health follow-up for discharged patients, as it may develop into geriatric depression (code CNCHF2).

“There are patients who are mentally weak, and there are also many who develop geriatric depression, so we continue to work with certified psychiatric nurses, and for those who really fall into a depressed state, we may consult with a psychiatrist. Also, after discharge from the hospital, we have a home nursing station, so we request a home nurse to visit the patients regularly and provide mental support for them.” (CNCHF2).

#### Share the patient’s prognosis and future wishes with multiple professionals

This category includes six sub-categories: (12a) Involve patients who have heart failure and need NPPV from the time of hospitalization, or who are recognized by a physician that the patient needed further consultation with GCNSs/CNCHFs; (12b) Understand the patient’s future and intentions through conferences with various professionals; (12c) Share the patient’s wishes among nurses and collaborate with the physician to realize them; (12d) Share with other nurses and practice the future outlook and policies of nursing care; (12e) Connect patients with multi-professionals to make home care possible by focusing on the patient’s life wishes after discharge from the hospital; and (12f) Provide patients and families with lifestyle guidance to maintain life after discharge by a multidisciplinary team including the outpatient department.

Specialized nurses commonly understand the patient's future with the team members who are involved with the patient (code GCNS5).

“I think that being involved intentionally means being able to see what is going to happen to the patient in the future. If nurses can't see that, nurses are just following what the ward staff tells them. When a team is formed, of course the nurses are involved, but I think it is important to have someone who is centered among the team members. So, if a patient has heart failure, the heart failure team will work as a team, and if a patient has heart diseases, the heart team will work as a team, and if everyone can see the whole picture of the patient, the team must be able to see what will happen to this patient in order to make adjustments.” (GCNS5).

#### Engage in ACP from early stages through several conversations with patients and their families

The final category includes four sub-categories: (13a) Perform ACP as early as possible before the condition transitions to advanced heart failure; (13b) Capture the topic of conversation with the patient including what the patient says and the atmosphere before linking it to ACP; (13c) Approach the patient in a timeous manner until the patient adequately understands their medical condition; and (13d) Confirm the thoughts and wishes of patients with their families and work collectively to realize them.

Specialized nurses talk to the patients about their thoughts and wishes as early as possible before the transition to advanced heart failure or pump failure, considering the risk of sudden death (code GCNS4). They also listen to the thoughts and feelings of patients and their families, consult with them, and involve them in making decisions that they can consider and have no regrets about (code CNCHF2).

“He/she was already in a bad condition. But he/she was able to communicate with us, and he/she asked us to let him/her go home as soon as possible. He/she said, ‘If I don’t go home now, I don’t know if I will be able to recognize myself when I get home, so please let me go home soon.’ But a few days later, he/she got really bad and went back to the CCU (critical care unit), and he/she passed away about a week later. I felt that I should have talked about the family’s thoughts and the patient’s wishes more in advance, and there was a situation recently that made me regret it” (GCNS4).

“The most important thing is to listen to the thoughts of the patients and their families. After listening to them, I think it’s better to consult with them and let them make their own decisions based on their own thoughts. I think it would be better if they can make a decision that neither the patient nor the family will regret, rather than just because the doctor said so or the nurse said so.” (CNCHF2).

## Discussion

Older patients during the onset of chronic heart failure tend to be unstable because their blood pressure most likely increases during this period, consequently exacerbating their heart failure [4, 16]. This is reflected in the following categories: the GCNSs/CNCHFs in our study stated that they *provide thorough acute care by a multidisciplinary team to alleviate dyspnea (1)* (the Italicized sentence indicates categories; the Arabic number delineates the order of appearance in the Results section) and that they *assess psychiatric symptoms and use a suitable environment to perform treatment (2)* to provide acute care. It is also important to understand the course of the disease at the onset of chronic heart failure, which indicates the beginning of the treatment. Mishel [17] defined uncertainty as the inability to determine the meaning of illness-related events. Furthermore, Mishel [17] showed that managing the uncertainty associated with an illness and its treatment is essential for adaptation. This suggests that it is important to understand the course of the disease to manage the uncertainties and adapt to the disease at the onset of chronic heart failure. Moreover, the GCNSs/CNCHFs in the present study also emphasized that they *explain the progression of heart failure with the doctor (3)* during the onset period.

Early implementation of ACP is recommended for older patients with heart failure [18–20]. Furthermore, at the initiation of ACP, Mullick et al. [19] recommend starting with general questions to avoid causing anxiety, while LeMond et al. [21] recommend starting a conversation and establishing goals. As shown by the category, *build a trusting relationship with the patient and family and implement ACP early during the patient's recovery (4)*, GCNSs/CNCHFs specifically built trust relationships through communication with patients and their families and implemented ACP from the onset of chronic heart failure. Mullick et al. [19] also suggested that worsening symptoms could trigger ACP. In line with this, it was clear that GCNSs/CNCHFs considered ACP should be implemented when the patient can converse following gradual recovery, as reported by GCNS3. Particularly, this is because ACP is implemented at the onset of chronic heart failure rather than after symptom recovery. Also, the implementation of ACP and the realization of the various lifestyles desired by older patients requires the restructuring of life and the introduction of social resources for activities of daily living. Similarly, the American Heart Association [16] also recognized this regarding the complexity of heart failure management and the coordination of other health and social services. As it was clarified in this study that GCNSs/CNCHFs *involve multiple professions to help patients to achieve their desired life (5)*, it is necessary to link patients with multidisciplinary professionals from the onset of chronic heart failure to fulfill the patient's wishes.

Regarding decompensation in a patient’s disease trajectory, European Guidelines indicated that an improvement in quality of life and psychological, social, and spiritual well-being should be emphasized [4]. It is highly unlikely that the intentions of patients will not be confirmed during this next advanced period. In this regard, we believe that the category *perform ACP always in collaboration with multiple professionals (6*) was proposed regardless of whether the patient is admitted for the first time or readmitted. Furthermore, a patient’s wishes can change with time. Thus, it is necessary to confirm whether the patient's initial intentions have changed. Although patients are expected to gradually decline in their function after decompensation, patients can continue living at home for a long time by preventing acute deterioration. Therefore, GCNSs/CNCHFs *provided lifestyle guidance according to patients' feelings, so that they can continue living at home after discharge from the hospital (7*).

During advanced chronic heart failure, therapy refers to the increased intensity of palliative care when standard therapies (including oral therapies) begin to fail [3]. When *providing palliative and acute care in parallel with multiple professions (8)*, acute care is also provided to palliate symptoms during this period [3]. This characterizes end-of-life care for older patients with heart failure. Conversely, regarding cases with very severe symptoms, GCNSs/CNCHFs *achieve end-of-life care at home through multidisciplinary cooperation (9)*. Thus, our findings indicated that end-of-life care at home is possible during this period, as GCNSs/CNCHFs play a key role in multidisciplinary collaboration and coordinate the discharge of patients.

Older adults can live with dignity [22]. Furthermore, nursing uniquely functions to assist in ways that contribute to a peaceful death [23]. An advanced state refers to a time that is near death. As indicated by our findings, GCNSs/CNCHFs *provide basic nursing care to the patient and family until the moment of death (10)*. Thus, the current study found that the basics of nursing care are required and provided even during the dying period.

To realize the implementation of ACP, the key element of end-of-life care for older patients with heart failure, this study also identified that GCNSs/CNCHFs *share the patient’s prognosis and future wishes with multiple professionals (12)* without being limited by the time period. In addition, they *engage in ACP from early stages, through several conversations with patients and their families (13)*. Moreover, the GCNSs/CNCHFs *provide concurrent acute and palliative care as well as psychological support to alleviate physical and mental symptoms (11)*; thus, they provide a multifaceted approach including physical and psychological aspects at the same time, without separating them.

### Strengths and limitations of the work

The participants of this study were GCNSs/CNCHFs in Japan. Thus, future studies should include specialized nurses from other countries with aging populations to confirm the generation of new data. However, the findings were meaningful because Japan is an aging society and has extensive experience in caring for older patients with heart failure. Some practices identified in this study could also be implemented by general nurses.

## Conclusions

In a complex trajectory, GCNSs/CNCHFs consistently provide concurrent acute care, palliative care, and psychological support to alleviate physical and mental symptoms throughout the different stages of heart failure in older patients. Particularly, specialized nurses connected patients with multiple professionals by sharing predictions of the patient’s situation and wishes for the future. Furthermore, they engaged in ACP from early stages, through several conversations with patients and their families.

The characteristics of nursing practices by disease stage of heart failure were also identified in the present study. In the period of onset, the approach involved providing acute care thoroughly by multidisciplinary staff, promoting patients’ understanding of the disease progress, and initiating ACP. In the period of decompensation, ACP was provided by multidisciplinary staff in anticipation of the next period, while lifestyle guidance was provided to ensure the patient continued living at home. In the period of advanced chronic heart failure, GCNSs/CNCHFs provided basic nursing care until the moment of death. Simultaneously, specialized nurses worked with the multidisciplinary team to palliate symptoms by ensuring acute care while increasing the emphasis on palliation. Moreover, end-of-life care at home could be implemented in some cases through multidisciplinary cooperation.

## Data Availability

The datasets generated and analyzed during the current study are not publicly available due the ethics approval body does not allow it but are available from the corresponding author on reasonable request.

## Abbreviations

ACP: Advance care planning
CNCHF: Certified nurses in chronic heart failure
CNS: Certified nurse specialist
GCNS: Certified nurse specialists in gerontology
IVH: Intravenous hyperalimentation
NPPV: Non-invasive positive pressure ventilation
NYHA: New York heart association

## References

[1] World Heart Federation. Roadmap for heart failure. https://world-heart-federation.org/cvd-roadmaps/whf-global-roadmaps/heart-failure/. Accessed 27 Aug 2021.

[2] Shimokawa H, Miura M, Nochioka K, Sakata Y (2015). Heart failure as a general pandemic in Asia. Eur J Heart Fail.

[3] Allen LA, Stevenson LW, Grady KL, Goldstein NE, Matlock DD, Arnold RM (2012). Decision making in advanced heart failure: a scientific statement from the American heart association. Circ.

[4] McDonagh TA, Metra M, Adamo M, Gardner RS, Baumbach A, Böhm M (2021). 2021 ESC Guidelines for the diagnosis and treatment of acute and chronic heart failure: Developed by the Task Force for the diagnosis and treatment of acute and chronic heart failure of the European Society of Cardiology (ESC) With the special contribution of the Heart Failure Association (HFA) of the ESC. Eur Heart J.

[5] Gelfman LP, Kavalieratos D, Teuteberg WG, Lala A, Goldstein NE (2017). Primary palliative care for heart failure: what is it? How do we implement it?. Heart Fail Rev.

[6] Raat W, Smeets M, Vandewal I, Broekx L, Peters S, Janssens S (2021). Cardiologists’ perceptions on multidisciplinary collaboration in heart failure care - a qualitative study. BMC Health Serv Res.

[7] Uchmanowicz I, Lisiak M, Lelonek M, Jankowska EA, Pawlak A, Jaroch J (2020). A curriculum for heart failure nurses: an expert opinion of the section of nursing and medical technicians and the heart failure working group of the polish cardiac society. Kardiol Pol.

[8] Sato Y, Kuragaichi T, Nakayama H, Hotta K, Nishimoto Y, Kato T (2022). Developing multidisciplinary management of heart failure in the super-aging society of Japan. Circ J.

[9] Song P, Tang W (2019). The community-based integrated care system in Japan: Health care and nursing care challenges posed by super-aged society. BioSci Trends.

[10] Kuragaichi T, Kurozumi Y, Ohishi S, Sugano Y, Sakashita A, Kotooka N (2018). Nationwide survey of palliative care for patients with heart failure in Japan. Circ J.

[11] Flick U. An introduction to qualitative research. 7th ed. London: Sage; 2022.

[12] Hjelmfors L, Strömberg A, Friedrichsen M, Mårtensson J, Jaarsma T (2014). Communicating prognosis and end-of-life care to heart failure patients: a survey of heart failure nurses’ perspectives. Eur J Cardiovasc Nurs.

[13] National Health Service. What end of life care involves: End of life care; 2018. https://www.nhs.uk/conditions/end-of-life-care/what-it-involves-and-when-it-starts/.

[14] Hsieh HF, Shannon SE (2005). Three approaches to qualitative content analysis. Qual Health Res.

[15] Elo S, Kyngäs H (2008). The qualitative content analysis process. J Adv Nurs.

[16] Heidenreich PA, Bozkurt B, Aguilar D, Allen LA, Byun JJ, Colvin MM (2022). 2022 AHA/ACC/HFSA Guideline for the management of heart failure: a report of the American College of Cardiology/American Heart Association joint committee on clinical practice guidelines. Circ.

[17] Mishel MH (1988). Uncertainty in illness. Image J Nurs Sch.

[18] Martina D, Lin CP, Kristanti MS, Bramer WM, Mori M, Korfage IJ (2021). Advance care planning in Asia: a systematic narrative review of healthcare professionals’ knowledge, attitude, and experience. JAMDA.

[19] Mullick A, Martin J, Sallnow L (2013). An introduction to advance care planning in practice. BMJ.

[20] Tokunaga-Nakawatase Y, Ochiai R, Sanjo M, Tsuchihashi-Makaya M, Miyashita M (2020). Perceptions of physicians and nurses concerning advanced care planning for patients with heart failure in Japan. Ann Palliat Med.

[21] LeMond L, Camacho SA, Goodlin SJ (2015). Palliative care and decision making in advanced heart failure. Curr Treat Options Cardiovasc Med.

[22] United Nations. Principles for older persons. https://www.principles-older-persons. http://ohchr.org/en/instruments-mechanisms/instruments/united-nations.

[23] Henderson V (1977). The concept of nursing. J Adv Nurs.

